# *Clostridium butyricum* Supplementation Reduces Diarrhea in Preweaning Calves by Modulating Fecal Short-Chain Fatty Acids and Gut Microbiota

**DOI:** 10.3390/microorganisms13091993

**Published:** 2025-08-27

**Authors:** Peiyun Gao, Shaoyang Pang, Qianqian Wang, Yaqin Tang, Qiuyan Li, Wenju Zhang, Cunxi Nie, Xiaoling Ma, Junli Niu

**Affiliations:** 1 Animal Nutrition and Feed Science, College of Animal Science and Technology, Shihezi University, Shihezi 832000, China; 13654656106@163.com (P.G.); 15251239380@163.com (S.P.); 15864653585@163.com (Q.W.); t892585470@sina.com (Y.T.); qiuyanli2025@outlook.com (Q.L.); zhangwj1022@sina.com (W.Z.); niecunxi@shzu.edu.cn (C.N.); 2 Laboratory and Equipment Management Division, Shihezi University, Shihezi 832000, China

**Keywords:** *Clostridium butyricum*, dairy calves, growth performance, diarrhea, short-chain fatty acids, gut microbiota

## Abstract

This study investigated the effects of dietary supplementation with varying doses of *Clostridium butyricum* (*C. butyricum*) on growth performance, diarrhea incidence, serum biochemical parameters, fecal short-chain fatty acids (SCFAs), and fecal microbiota in preweaning Holstein calves. Forty healthy newborn Holstein heifer calves with comparable birth weights were randomly assigned to four groups (control, 1 g/d supplementation, 3 g/d supplementation, and 5 g/d supplementation) for a 60-day trial. Growth parameters, diarrhea incidence, serum immunoglobulins (IgA, IgG, IgM), cytokines (IL-1β, TNF-α), antioxidant indicators (T-AOC, MDA), fecal short-chain fatty acids (SCFAs), and microbial composition were measured to evaluate the effects of *C. butyricum*. The results indicated that calves in the 5 g/d group exhibited a significantly higher average daily gain (ADG) compared with the control group (804.67 vs. 701.67 g/d, *p* < 0.05), with no significant differences in feed intake observed among groups (*p* > 0.05). During Days 22–42, the diarrhea incidence in the 5 g/d group was 7.74% lower than that in the control group (*p* < 0.05). This group exhibited significantly elevated serum IgM levels (Day 42, *p* < 0.05) and reduced IL-1β concentrations (Day 42, *p* < 0.05). Additionally, total antioxidant capacity (T-AOC) was significantly enhanced (Days 42 and 60, *p* < 0.05), while malondialdehyde (MDA) content was significantly decreased (Days 21 and 42, *p* < 0.05). At Day 42, fecal propionate and butyrate concentrations were significantly elevated in the 5 g/d group (*p* < 0.05), while the relative abundances of *Bacteroides*, *Acidaminococcus*, *Bifidobacterium*, *Olsenella*, *Faecalitalea*, and *Ruminococcus* were significantly increased (*p* < 0.05). The increase in these short-chain fatty acids and beneficial bacteria contributes to improved intestinal health and thus helps alleviate diarrhea. These findings indicate that supplementing preweaning calves’ milk with 5 g/d of *C. butyricum* significantly enhances growth performance and intestinal health. This provides evidence for the use of *C. butyricum* as a natural alternative to antibiotics in calf rearing.

## 1. Introduction

Calf rearing constitutes a critical phase in high-quality dairy farming, forming the foundation for lifetime production performance in lactating cows. Moreover, it ensures herd stability and generates greater economic benefits. Therefore, ensuring the healthy growth of calves represents not only a primary objective in farm management but also serves as the cornerstone for the sustainable development of dairy operations. During early development, calves undergo their most rapid somatic growth phase, characterized by accelerated maturation of organ functions, enhanced immune system development, and high physiological plasticity. Environmental or nutritional interventions during this phase may substantially alter the animals’ subsequent growth trajectories. Since the 20th century, antibiotics have been widely applied as feed additives with growth-promoting properties in livestock and poultry production. However, indiscriminate antibiotic use has caused a range of negative consequences, including enhanced drug resistance in animals, drug residues in products, and food safety concerns [1]. Therefore, since 2006, the European Union, United States, and China have successively issued regulations prohibiting the use of antibiotics as feed additives [2].

Probiotics, including *Lactobacillus*, yeast, and *Bacillus* species, have emerged as promising antibiotic alternatives. They not only prevent intestinal microbiota disruption but also promote microbial homeostasis by enhancing multiple metabolic pathways, thereby maintaining normal gut function [3]. *Lactobacillus* primarily inhibits pathogenic bacteria by producing lactic acid, which lowers the pH level [4]. However, the probiotic function of *Bacillus* does not typically involve producing large quantities of short-chain fatty acids [5]. *Clostridium butyricum*, an anaerobic Gram-positive bacillus inhabiting the gastrointestinal tract, produces metabolites including butyrate, digestive enzymes, and bacteriocins. It has attracted significant attention due to its probiotic properties, such as imparting anti-inflammatory effects, enhancing immunity, and modulating the gut microbiota [6,7]. Research on the application of *Clostridium butyricum* in ruminant production remains relatively scarce compared with studies in monogastric animals such as poultry and swine. This is potentially due to the complexity of the rumen ecosystem and the fact that research has focused more on postweaning rumen function than on preweaning intestinal health. The mechanisms linking *C. butyricum* supplementation to changes in the gut microbiota, short-chain fatty acid production, and the immune system in this population have not yet been fully elucidated. Therefore, we hypothesized that dietary supplementation with *C. butyricum* would ameliorate diarrhea and enhance growth performance in a dose-dependent manner in preweaning calves. This trial investigated the effects of varying supplemental doses of *C. butyricum* on growth performance and health in preweaning calves. The study concurrently examined underlying mechanisms to provide foundational theoretical support for its application in ruminant production systems.

## 2. Materials and Methods

### 2.1. Strains

*Clostridium butyricum* (*C. butyricum*) was supplied by Vland Biotechnology Co., Ltd. (Qingdao, China). The viable bacterial preparation was provided as freeze-dried powder containing 1 × 10^9^ CFU/g.

### 2.2. Animals and Diet

The animal care protocol was approved by the Animal Welfare Committee of Shihezi University (Shihezi, China) (Ethics No. A2023-616).

Forty healthy newborn Holstein heifer calves with comparable birth weights (38.16 ± 3.73 kg) were randomly assigned to four groups, with 10 calves per group. The sample size was determined with reference to the design of previous similar studies that successfully detected significant differences at close sample sizes [8,9]. The control group received the basal diet, while the 1 g, 3 g, and 5 g groups were fed the basal diet supplemented with 1 g/d, 3 g/d, and 5 g/d of *C. butyricum* per calf, respectively. All of the calves were born on the same farm in the summer. They were vaccinated and dewormed after birth. The trial spanned 60 days. Within 1 h postpartum, each calf received 4 L of high-quality colostrum and was subsequently housed in individual hutches. Milk feeding strictly adhered to the farm’s protocol, with pasteurized milk provided daily at 06:00 and 18:00. Feeding amounts were as follows: Week 1: 5 L/head; Week 2: 6 L/head; Week 3: 7 L/head; Weeks 4–7: 8 L/head. From Day 50, the milk allowance was incrementally reduced by 2 L every two days until complete weaning at Day 60. Calves received starter feed from Day 7 and were provided alfalfa hay ad libitum from Day 50. Throughout the trial, all calves had ad libitum access to feed and water. Hutches were regularly cleaned and disinfected following standard farm protocols. *C. butyricum* was thoroughly blended into milk immediately prior to feeding to ensure complete ingestion by calves. The study was conducted with complete randomization to mitigate potential bias during the subsequent sample collection and analysis procedures. The randomization grouping process did not impede the calves based on birth weight or other factors. The ingredients and nutritional composition of the starter feed and alfalfa hay are detailed in Table 1.

### 2.3. Sample Collection

Daily feed intake and refusals of starter feed and alfalfa hay were recorded. Samples of both feeds were collected for nutrient analysis.

On Days 21, 42, and 60, six calves per group were randomly selected pre-morning feeding. Blood samples (10 mL) were collected from the jugular vein using sodium heparin vacuum tubes. After clotting for 30 min at room temperature, the samples were centrifuged at 3000× *g* for 15 min. Plasma was aliquoted into sterile tubes and stored at −20 °C.

For fecal sampling, six calves per group were similarly selected at identical timepoints. Samples were collected using sterile rectal sleeves, immediately transferred to sterile cryovials, snap-frozen in liquid nitrogen, and stored at −80 °C.

### 2.4. Analysis of Growth Performance and the Incidence of Diarrhea

Average daily gain (ADG) was calculated from body weights recorded on Days 1, 21, 42, and 60. Dry matter intake (DMI) of milk and starter feed was monitored throughout the trial. Starter feed samples were analyzed for dry matter (DM; AOAC 930.15), crude protein (CP; AOAC 976.05), and ether extract (AOAC 4.5.05), following AOAC International standards [10,11]. Neutral detergent fiber (NDF) and acid detergent fiber (ADF) contents were determined as per Van Soest et al. [12].

Fecal consistency was scored as 1 = normal; 2 = soft to unformed; 3 = unformed to liquid; 4 = liquid with mucous and blood-tinged; 5 = liquid with mucous and frankly bloody. Diarrhea was defined as a score > 3 [13]. Incidence was calculated using

Diarrhea incidence (%) = (Number of diarrheic calves × Days with diarrhea)/(total calves × trial days) × 100%.

### 2.5. Serum Analysis

Serum concentrations of immunoglobulin M (IgM), immunoglobulin A (IgA), immunoglobulin G (IgG), tumor necrosis factor-α (TNF-α), interleukin-2 (IL-2), interleukin-1β (IL-1β), total antioxidant capacity (T-AOC), and malondialdehyde (MDA) were quantified using commercial ELISA kits (Shanghai Enzyme-linked Biotechnology, Shanghai, China).

### 2.6. 16 S rRNA Gene Sequencing and Analysis

Total bacterial DNA was extracted from fecal samples using the TIANamp Stool DNA Kit (Tiangen, Beijing, China). The V3–V4 hypervariable region of the 16S rRNA gene was amplified with the primers 338F (5′-ACTCCTACGGGAGGCAGCA-3′) and 806R (5′-GGACTACHVGGGTWTCTAAT-3′). Sequencing was performed on an Illumina MiSeq platform (Illumina, San Diego, CA, USA) by Majorbio Bio-Pharm Technology Co., Ltd. (Shanghai, China).

Raw sequences were processed in QIIME2. High-quality reads were clustered into operational taxonomic units (OTUs) at 97% similarity using UCLUST (v7.1). Taxonomic assignment against the Greengenes 13.5 database was performed with PyNAST. Alpha diversity indices (Chao1, ACE, Shannon, Simpson) and beta diversity (weighted UniFrac PCoA) were analyzed. Differential taxa identification employed the linear discriminant analysis effect size (LEfSe). Raw sequencing data are deposited in the NCBI SRA under accession PRJNA1294751.

### 2.7. Targeted SCFA Metabolomics

#### 2.7.1. Sample Preparation

Fecal samples (≈0.5 g) were homogenized in 1.5 mL microtubes with 500 μL of distilled water and 100 mg of 0.1 mm glass beads (1 min bead beating). After centrifugation (12,000× *g*, 4 °C, 10 min), 200 μL of the supernatant was transferred to a new tube. Then, 100 μL of 15% phosphoric acid (Sigma-Aldrich, Saint Louis, MO, USA), 20 μL of 4-methylvaleric acid internal standard (375 μg/mL; Sigma-Aldrich), and 280 μL diethyl ether (Merck, Darmstadt, Germany) were added. The mixture was vortexed (1 min) and centrifuged under identical conditions. The ether phase was transferred to a GC-MS vial.

#### 2.7.2. GC-MS Analysis

Gas chromatography conditions

Chromatographic separation employed a Trace 1310 GC system (Thermo Fisher Scientific, Waltham, MA, USA) with an Agilent HP-INNOWAX capillary column (30 m × 0.25 mm ID × 0.25 μm). Helium carrier gas was maintained at 1 mL/min (constant flow). Samples were injected in split mode (10:1) with 1 μL volume at 250 °C. Ion source and transfer line temperatures were 300 °C and 250 °C, respectively. The oven program was initiated at 90 °C then ramped to 120 °C (10 °C/min), 150 °C (5 °C/min), and finally 250 °C (25 °C/min; held for 2 min).

Mass spectrum conditions

Mass spectrometry detection was conducted using an ISQ 7000 system (Thermo Fisher Scientific, USA) in electron impact (EI) ionization mode. Analyses employed selected ion monitoring (SIM) with 70 eV electron energy.

### 2.8. Statistical Analysis

Data were analyzed by two-way ANOVA using SPSS (version 27.0). The results are presented as least-squares means ± SEM. Statistical significance was defined as *p* < 0.05.

## 3. Results

### 3.1. Growth Performance and Diarrhea Incidence

As shown in Table 2, no significant differences occurred in initial body weight among groups (*p* > 0.05). Calves supplemented with 5 g/d *C. butyricum* exhibited significantly higher average daily gain (ADG) versus the controls (*p* < 0.05). Dry matter intake (DMI) of starter feed and total feed showed no significant intergroup differences (*p* > 0.05), and the gain-to-feed ratio remained comparable across treatments. Supplementation with 5 g/d *C. butyricum* significantly reduced diarrhea incidence at 42 days relative to the controls (*p* < 0.05).

### 3.2. Plasma Immunoglobulin, Cytokine, and Antioxidant Index Levels

As shown in Table 3, supplementation with *C. butyricum* had no significant effect on plasma IgA or IgG levels at any sampled time point (*p* > 0.05). However, calves in the 5 g *C. butyricum* group displayed significantly higher serum IgM concentrations than the control group at 42 days of age (*p* < 0.05). Concurrently, plasma IL-1β levels in the 5 g group were markedly lower than those in the control group at 42 days (*p* < 0.05).

No significant differences in IL-2 or TNF-α levels were detected among the groups at any measured time point (*p* > 0.05). The 5 g *C. butyricum* group exhibited significantly lower serum malondialdehyde (MDA) levels at both 21 and 42 days of age compared with the controls (*p* < 0.05). Additionally, total antioxidant capacity (T-AOC) was markedly enhanced in the 5 g group at 42 and 60 days (*p* < 0.05).

### 3.3. Comparative Analysis of Short-Chain Fatty Acids

**Figure 1:**
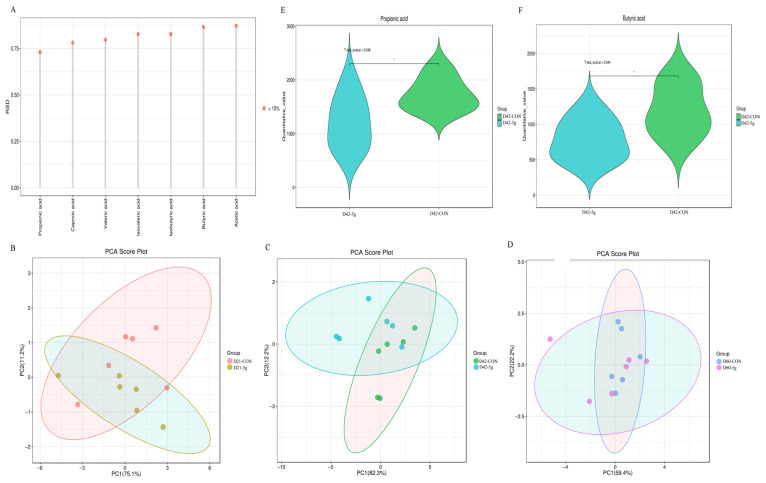
Multivariate statistical analysis of short-chain fatty acid. (**A**) The stability of short-chain fatty acid in QC samples. (**B**) PCA score plot of SCFAs in rectal samples of calves at Day 21. (**C**) PCA score plot of SCFAs in rectal samples of calves at Day 42. (**D**) PCA score plot of SCFAs in rectal samples of calves at Day 60. (**E**) Propionic acid concentrations in rectal samples of calves at Day 42. (**F**) Butyric acid concentrations in rectal samples of calves at Day 42.

The relative standard deviations (RSDs) for all targeted metabolites remained below 15% (Figure 1A), confirming the analytical method’s stability and reliable quantification. At 21, 42, and 60 days, Principal Components 1 and 2 accounted for 75.1% and 11.2% (Figure 1B), 82.3% and 12.2% (Figure 1C), and 59.4% and 22.2% (Figure 1D) of the total variance, respectively.

Table 4, Table 5 and Table 6 illustrate fecal short-chain fatty acid content in control calves and calves fed with *C. butyricum* for 21, 42, and 60 days, respectively. The fecal propionic acid (Figure 1E) and butyric acid (Figure 1F) concentrations were significantly elevated in the 5 g/d group compared with the controls on Day 42 (*p* < 0.05) (Table 5). No significant differences in short-chain fatty acid levels were observed among groups at 21 and 60 days (*p* > 0.05) (Table 4 and Table 6).

### 3.4. Rectal Microbial Changes

**Figure 2:**
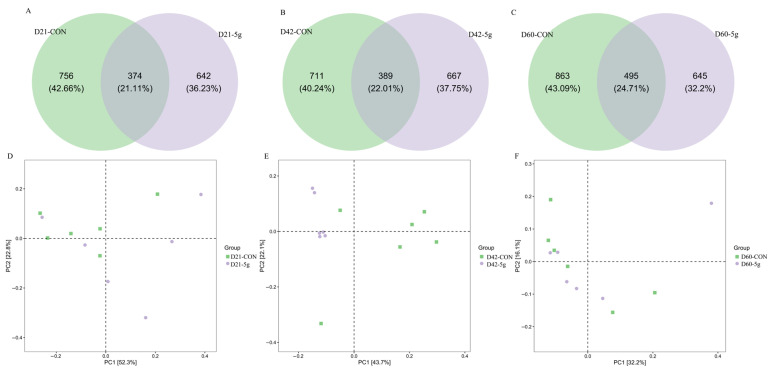
Venn diagram and principal coordinate analysis in rectal samples of calves. In the Venn diagram, green represents OTUs in the control group and purple represents OTUs in the group fed 5 g/d *C. butyricum*. The overlap represents OTUs present in both groups. (**A**) Venn diagram in rectal samples of calves at Day 21. (**B**) Venn diagram in rectal samples of calves at Day 42. (**C**) Venn diagram in rectal samples of calves at Day 60. (**D**) Principal coordinate analysis in rectal samples of calves at Day 21. (**E**) Principal coordinate analysis in rectal samples of calves at Day 42. (**F**) Principal coordinate analysis in rectal samples of calves at Day 60.

At Day 21 (d21), the control (CON) and 5 g *C. butyricum* groups exhibited total observed OTU counts of 1139 and 1016, respectively, with 374 shared OTUs representing 21.11% of the cumulative microbial diversity (Figure 2A). By Day 42 (d42), OTU counts shifted to 1100 (control) and 1056 (5 g group), with 389 overlapping OTUs constituting 22.01% of the total taxonomic units (Figure 2B). At Day 60 (d60), the control group demonstrated increased OTU richness (1358), while the 5 g group showed 1140 OTUs, sharing 495 core microbial taxa that accounted for 24.71% of the combined OTU repertoire (Figure 2C).

**Figure 3:**
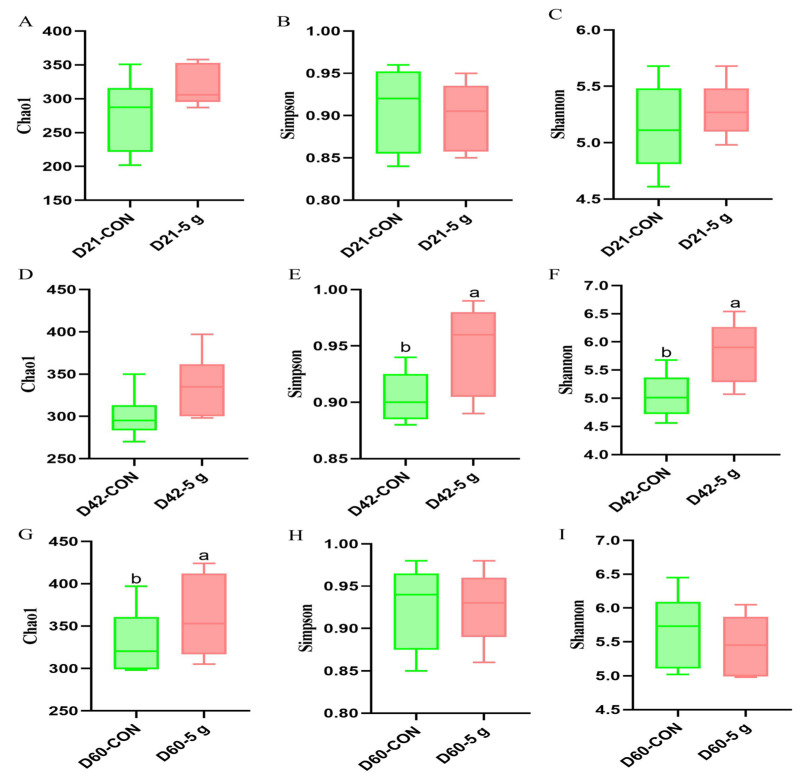
Richness and diversity of feces of calves fed with *C. butyricum*. ^a,b^ Indicates a significant difference between groups (*p* ≤ 0.05). (**A**) Chao1 index in rectal samples of calves at Day 21. (**B**) Simpson index in rectal samples of calves at Day 21. (**C**) Shannon index in rectal samples of calves at Day 21. (**D**) Chao1 index in rectal samples of calves at Day 42. (**E**) Simpson index in rectal samples of calves at Day 42. (**F**) Shannon index in rectal samples of calves at Day 42. (**G**) Chao1 index in rectal samples of calves at Day 60. (**H**) Simpson index in rectal samples of calves at Day 60. (**I**) Shannon index in rectal samples of calves at Day 60.

For the 21-day-old calves, the contribution rates of PC1 and PC2 were 52.3% and 22.8%, respectively (Figure 2D). For the 42-day-old calves, the contribution rates of PC1 and PC2 were 43.7% and 22.1%, respectively (Figure 2E). For the 60-day-old calves, the contribution rates of PC1 and PC2 were 32.2% and 16.1%, respectively (Figure 2F). Additionally, at 42 days of age, the individual samples within the CON and 5 g groups were closely clustered, and the two groups were distinctly separated, indicating significant differences in the fecal microbiota (Figure 2D). At 42 days of age, the Simpson and Shannon indices of fecal microbiota in the 5 g group were significantly higher than those in the control group (*p* < 0.05) (Figure 3E,F). At d60, the Chao1 index in the 5 g group was significantly higher than that in the control group (*p* < 0.05) (Figure 3G). At other ages, there were no significant differences in alpha diversity among the treatment groups (*p* > 0.05) (Figure 3).

**Figure 4:**
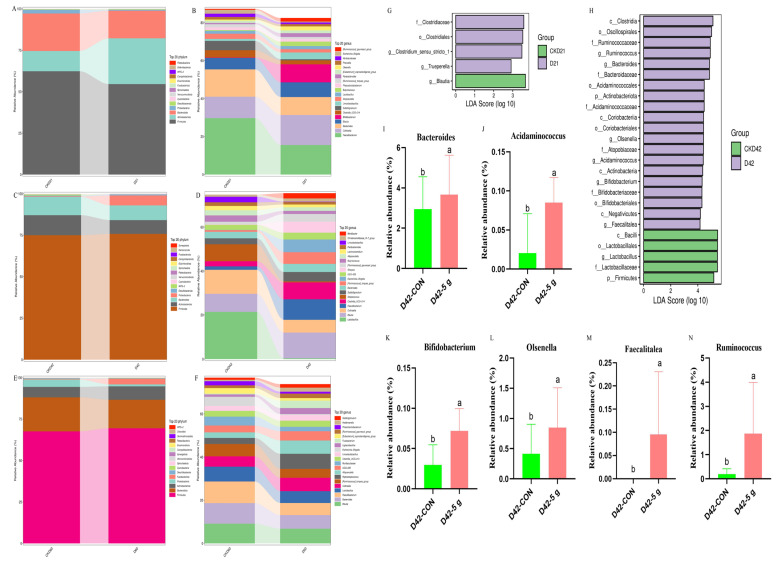
Microbial community composition in rectal samples of calves. ^a,b^ Indicates a significant difference between groups (*p* ≤ 0.05). (**A**) Bacterial composition at the phylum level at Day 21. (**B**) Bacterial composition at the genus level at Day 21. (**C**) Bacterial composition at the phylum level at Day 42. (**D**) Bacterial composition at the genus level at Day 42. (**E**) Bacterial composition at the phylum level at Day 60. (**F**) Bacterial composition at the genus level at Day 60. (**G**) LEfSe analysis in rectal microbiota of calves at Day 21. The graph shows that green represents the control group and purple represents the group fed 5 g/d of *C. butyricum*. Each horizontal bar represents a species that is statistically significantly different from the others. The length of each bar corresponds to the LDA score. Higher scores indicate that the species contributes more to differentiating between the two groups. (**H**) LEfSe analysis in the rectal microbiota of calves at Day 42. The same description applies to Figure 4G. (**I**) The relative abundance of *Bacteroides* in the rectal microbiota of calves at Day 42. (**J**) The relative abundance of *Acidaminococcus* in the rectal microbiota of calves at Day 42. (**K**) The relative abundance of *Bifidobacterium* in the rectal microbiota of calves at Day 42. (**L**) The relative abundance of *Olsenella* in the rectal microbiota of calves at Day 42. (**M**) The relative abundance of *Faecalitalea* in the rectal microbiota of calves at Day 42. (**N**) The relative abundance of *Ruminococcus* in the rectal microbiota of calves at Day 42.

At the phylum level, fecal microbiota in both the CON and 5 g/d groups were predominantly composed of *Bacteroidota*, *Actinobacteriota*, and *Firmicutes* at 21, 42, and 60 days (Figure 4A,C,E). At 21 days, *Bacteroides*, *Collinsella*, and *Faecalibacterium* constituted the predominant genera (Figure 4B). By 42 days, the CON group was dominated by Collinsella, *Blautia*, and *Lactobacillus* (Figure 4D), whereas *Blautia*, *Faecalibacterium*, and *Clostridia*_UCG-014 predominated in the 5 g/d group. At 60 days, *Blautia*, *Bacteroides*, and *Faecalibacterium* emerged as the dominant genera in both groups (Figure 4F).

LEfSe analysis revealed distinct microbial compositions between groups (Figure 4G,H). At 21 days, the 5 g/d group exhibited significantly greater relative abundances of *Clostridium*_sensu_stricto_1 and Trueperella compared with the controls, whereas the control group showed higher Blautia abundance (Figure 4G). At 42 days, the 5 g/d group demonstrated significantly elevated relative abundances of *Bacteroides* (Figure 4I), *Acidaminococcus* (Figure 4J), *Bifidobacterium* (Figure 4K), *Olsenella* (Figure 4L), *Faecalitalea* (Figure 4M), and *Ruminococcus* (Figure 4N) versus the controls (Figure 4H). No significant compositional differences were observed between groups at 60 days.

## 4. Discussion

### 4.1. Effects of Clostridium butyricum on Growth Performance in Preweaning Calves

The health status of preweaning calves directly influences their subsequent development and adult production performance. Growth performance serves as a critical indicator for evaluating calf development [14], typically assessed through metrics including average daily gain (ADG), dry matter intake (DMI), and gain-to-feed ratio [15]. Current research on probiotic effects on livestock growth primarily focuses on lactic acid bacteria, Bacillus, and yeast. Studies involving *Clostridium butyricum* concentrate predominantly on monogastric animals such as chickens, rabbits, and swine, where it demonstrates beneficial effects on growth performance. Zhao et al. [16] demonstrated that dietary supplementation with *Clostridium butyricum* significantly increased body weight and average daily gain in 21-day-old broilers. Similarly, Yang et al. [17] reported enhanced ADG in broilers supplemented with *C. butyricum*, with more pronounced effects during later growth stages. Xue et al. [18] observed significantly increased DMI, ADG, and digestibility of DM, NDF, and ADF in goats receiving *C. butyricum* supplementation. Cai et al. [19] likewise documented elevated DMI, ADG, and improved digestibility of DM, NDF, and ADF in ruminants administered probiotics.

In this trial, supplementing preweaning calves’ milk replacer with *Clostridium butyricum* modestly improved average daily gain compared with controls, consistent with previous studies. This effect may be attributed to *C. butyricum*-derived metabolites such as butyrate and vitamins that enhance physiological development [20]. Concurrently, secreted proteases facilitate beneficial gut microbiota colonization, thereby improving nutrient digestion and absorption, and feed efficiency [21]. The limited growth response may relate to animal breed variations and dosage factors. However, some studies report no significant effects on diarrhea incidence or growth [22,23], with discrepancies potentially arising from differences in animal species, supplementation levels, or environmental conditions. Collectively, these findings suggest *C. butyricum* exerts moderate growth-promoting effects in preweaning calves.

### 4.2. Effects of Clostridium butyricum on Diarrhea Reduction in Preweaning Calves

At birth, calves possess an immature gastrointestinal microbial community and an underdeveloped immune system, rendering the gut ecosystem highly vulnerable to pathogenic disruption. A stable, healthy microbiota exerts long-term impacts on the host’s phenotypic traits and production performance [24]. Diarrhea represents a critical factor compromising the health of preweaning calves and constitutes a primary cause of mortality. Previous research has demonstrated that probiotics modulate intestinal microbial balance. By colonizing the gastrointestinal tract, they produce localized antimicrobial substances that inhibit pathogenic proliferation and promote beneficial microbiota establishment, thereby reducing diarrheal incidence [25,26]. Studies indicate that dietary *Clostridium butyricum* supplementation reduces diarrhea incidence in livestock and poultry to varying degrees [27]. Wu et al. [28] investigated the effect of *C. butyricum* on diarrhea in weaned piglets. Their results demonstrated significantly lower diarrhea incidence in piglets supplemented with *C. butyricum* compared with the controls during the nursing period. Reduced diarrhea incidence lowers direct costs for veterinary interventions and antibiotic usage while improving diarrheal management in calves. This prevention of associated appetite reduction mitigates production performance losses. Furthermore, it contributes to enhanced animal welfare and generates economic benefits for farms [29]. In this study, diarrhea incidence in control calves fluctuated between 7% and 36% during the trial, decreasing with advancing age. Diarrhea rates in *C. butyricum*-supplemented groups showed no significant difference from the controls during the first three weeks. However, the 5 g/d group exhibited significantly lower diarrhea incidence than the controls during Weeks 4–8, consistent with previous findings that early probiotic intervention demonstrates preventive efficacy against diarrhea. We postulate that reduced diarrhea incidence may be closely linked to enhanced immune function, improved gut barrier integrity, and altered gut microbiota composition. These mechanisms likely contributed to the observed growth performance improvements, consistent with the growth data obtained in this trial.

### 4.3. Effects of Clostridium butyricum on Serum Immune and Antioxidant Functions in Preweaning Calves

Antioxidant capacity and immune function represent two critical indicators reflecting an animal’s health status and disease resistance, enabling assessment of systemic antioxidant status and immune response levels. The liver plays a crucial role in regulating oxidative stress and immune responses in vivo. Immunoglobulins (including IgA, IgG, and IgM) are antibody-active proteins that mediate antiviral, antibacterial, and other immunological functions, primarily through antibody-secreting B lymphocytes. Research indicates that probiotics modulate various immune cells to enhance host immunity [30]. Similarly, Zhang et al. [31,32] demonstrated that dietary *C. butyricum* supplementation elevates serum immunoglobulin concentrations in weaned piglets and broilers. The present findings demonstrate that dietary *C. butyricum* supplementation enhanced serum IgA, IgM, and IgG concentrations throughout the preweaning period. This variation likely resulted from differential *C. butyricum* dosing levels.

Antioxidant capacity is essential for maintaining organismal homeostasis in animals. Under normal physiological conditions, endogenous antioxidant enzymes primarily scavenge free radicals to sustain dynamic equilibrium [33]. When animals experience endogenous or exogenous stimuli, antioxidant systems mobilize to scavenge free radicals, thereby maintaining redox homeostasis and preserving animal health. Total antioxidant capacity (T-AOC) serves as a key parameter for assessing systemic antioxidant capacity, while malondialdehyde (MDA) reflects the lipid peroxidation extent, indirectly indicating oxidative damage severity. Lower MDA concentrations denote stronger antioxidant capacity. This study measured these parameters to assess antioxidant status. Plasma T-AOC levels were significantly elevated in calves aged 22–60 days compared with the controls, while MDA levels were significantly reduced in calves aged 0–42 days. *C. butyricum* modulates the body’s antioxidant capacity primarily by activating key antioxidant signaling pathways [34] and enhancing the expression of antioxidant enzymes [35], thereby alleviating oxidative damage.

### 4.4. Effects of Clostridium butyricum on Intestinal SCFA Production in Preweaning Calves

Short-chain fatty acids (SCFAs), also termed volatile fatty acids, are produced through microbial fermentation of indigestible complex carbohydrates—such as dietary fiber and non-starch polysaccharides—in the gastrointestinal tract. They play pivotal roles in regulating immune responses, energy metabolism, and cellular proliferation [36]. Acetate, propionate, and butyrate represent the most abundant SCFAs [37]. Butyric acid is the preferred energy source for colonic epithelial cells or colon cells. An adequate supply of butyric acid efficiently nourishes colon cells, promoting their proliferation and normal function. This is crucial for maintaining intestinal barrier integrity [38]. Propionic acid serves as a substrate for gluconeogenesis in the liver. This process helps provide a stable supply of glucose to the body, regulating appetite and energy metabolism [39]. Therefore, increasing both butyric acid and propionic acid creates a healthier intestinal microenvironment. Sallem et al. [40] observed that multi-strain probiotics promote intestinal SCFA production. Li et al. [41] documented significantly increased fecal butyrate and total SCFA concentrations in domestic shorthair cats administered probiotic formulations. Zhang et al. [42] reported elevated SCFA levels in hyperlipidemic rats supplemented with Pediococcus acidilactici. Similarly, Jiang et al. [43] demonstrated significantly higher fecal acetate, propionate, and butyrate concentrations in neonatal Holstein calves receiving *Lactobacillus* supplementation. The results indicate that at 42 days, calves supplemented with 5 g/d *Clostridium butyricum* exhibited significantly higher fecal propionate and butyrate concentrations than the controls. This effect may stem from *C. butyricum* promoting beneficial microbial proliferation, thereby providing substrates for propionate- and butyrate-producing bacteria. Concurrently, *C. butyricum* likely inhibited enteric pathogens while stimulating intestinal epithelial proliferation and mucosal development, contributing to diarrhea alleviation [44]. These findings align with the reduced diarrhea incidence observed in the *C. butyricm*-supplemented group. Thus, 5 g/d *C. butyricum* supplementation enhances gastrointestinal health by elevating intestinal SCFA production.

### 4.5. Effects of Clostridium butyricum on the Gut Microbiota’s Structure and Microbial Abundance in Preweaning Calves

The composition of gastrointestinal microbiota fundamentally influences the health and growth performance of ruminants [45]. Dietary regimens during early development similarly shape microbial colonization in calves. Appropriate probiotic supplementation substantially benefits calf growth during this critical period [46]. Disruption of beneficial microbiota, coupled with the proliferation of pathogens or opportunistic bacteria, precipitates gastrointestinal disorders including diarrhea, enteritis, and functional impairment [47].

Multiple studies demonstrate that *Bacteroidota* represents one of the predominant phyla in bovine intestines [48,49], generally regarded as beneficial and protective. These microorganisms colonize and occupy substantial intestinal niches, limiting opportunities for pathogenic colonization. Furthermore, certain *Bacteroides* species modulate immune responses and exhibit anti-inflammatory properties [50]. Previous research suggests that Alistipes, a genus within *Bacteroidota*, may exert protective effects in the gut by producing anti-inflammatory fatty acids associated with host health [51]. The results of this experiment confirm the dominant status of *Bacteroidota* within the gastrointestinal microbial ecosystem and demonstrate significantly higher abundance in the fecal microbiota of calves supplemented with *C. butyricum* compared with the controls. Recent studies have demonstrated a synergistic, mutualistic “cross-feeding” relationship between *Clostridium butyricum* and *Bacteroidota* [52].

*Acidaminococcus*, a genus within the phylum *Verrucomicrobia*, comprises Gram-negative anaerobic cocci primarily colonizing human and animal intestines. These bacteria utilize amino acids (particularly glutamate) as their primary carbon source, producing metabolites including short-chain fatty acids [53,54]. Research indicates that increased abundance of *Acidaminococcus* may enhance protein digestion and nutrient absorption [55]. *Acidaminococcus* not only produces acetate and butyrate but also eliminates harmful metabolites during ruminal fermentation [56,57]. Through butyrate production, it promotes intestinal epithelial repair and enhances the host’s energy metabolism [54]. This study observed a higher relative abundance of *Acidaminococcus* in the fecal microbiota of calves supplemented with 5 g/d *C. butyricum* at Day 42 compared with the controls, aligning with previous findings.

Dietary supplementation with *C. butyricum* and particularly *Bifidobacterium* promotes the proliferation of butyrate-producing microorganisms and significantly influences microbial diversity and distinct community composition [58]. Research has found that feeding butyric acid bacteria to fattening pigs can increase the relative abundance of *Bifidobacterium* at the genus and species levels in fecal samples [59]. *Bifidobacterium* also enhances intestinal epithelial barrier function by downregulating claudin-2 expression [60]. Research indicates that *Bifidobacterium*, a dominant genus in fecal microbiota, is commonly used as a probiotic and exerts beneficial effects against various diseases, including rotavirus-associated diarrhea, antibiotic-associated diarrhea, and certain inflammatory bowel diseases [61]. This suggests that *Bifidobacterium* reduces diarrhea incidence by promoting butyrate production, enhancing intestinal barrier function, and modulating immune responses.

*Faecalitalea*, a genus within the *Firmicutes* phylum, ferments D-glucose, sucrose, D-mannose, and raffinose. It may protect intestinal barrier function through short-chain fatty acid (SCFA) production [62]. Moreover, as a butyrate producer, butyrate exerts potent anti-inflammatory effects that mitigate intestinal inflammation, thereby preventing inflammation-induced gut damage and fluid exudation. Consequently, *Faecalitalea* abundance is inversely correlated with calf diarrhea incidence [63]. The results of this experiment demonstrate significantly higher fecal abundances of *Bifidobacterium* and *Faecalitalea* in calves supplemented with 5 g/d *C. butyricum* compared with the control group.

*Olsenella*, a genus within the *Actinobacteria* phylum, comprises Gram-positive anaerobic bacilli commonly inhabiting mucosal environments including the oral cavity, intestine, and vagina [64]. This bacterium ferments carbohydrates to produce short-chain fatty acids (SCFAs) such as lactate and acetate. Research suggests that specific *Olsenella* strains participate in tryptophan metabolism to generate immunomodulatory metabolites (e.g., inosine). These strains enhance the efficacy of checkpoint blockade immunotherapy, significantly improving immune checkpoint inhibitor outcomes in four murine cancer models [65]. The results of this study demonstrate significantly higher fecal *Olsenella* abundance in calves supplemented with 5 g/d *C. butyricum* compared with the control group.

In the 5 g/d *C. butyricum* supplementation group, *Ruminococcus* constituted a dominant genus in calf fecal microbiota compared with the controls. Liu et al. [66] indicated that *Clostridium butyricum* can significantly increase the relative abundance of Ruminococcus in the intestines of obese mice and improve the composition of intestinal microbiota. *Ruminococcus*, a strictly anaerobic Gram-positive coccus belonging to the family *Lachnospiraceae*, actively ferments dietary fiber and polysaccharides [67]. Its primary metabolites include acetate, formate, and ethanol, though it does not produce butyrate. Certain strains metabolize mucins and human milk oligosaccharides (HMOs), influencing intestinal barrier integrity [68]. Research evidence indicates that *Ruminococcus* commonly inhabits the intestinal microbiota of humans and ruminants and may play a significant role in modulating intestinal inflammatory activity [69].

The results demonstrate a significantly higher relative abundance of *Ruminococcus* in the fecal microbiota of calves supplemented with 5 g/d *C. butyricum* at Day 42 compared with the control group. We therefore propose that supplementing *C. butyricum* during the preweaning period reduces diarrhea incidence by modulating gut microbial diversity. Despite the predominance of 16S rRNA sequencing in the field of prokaryotic research, its efficacy is limited in the analysis of eukaryotic microorganisms, such as fungi, and viral communities within complex samples. Secondly, DNA-based sequencing is unable to distinguish between surviving organisms and deceased organisms, nor can it differentiate between free environmental DNA and other sources of DNA. This limitation may have a significant impact on the biological interpretation of the results. Future studies should investigate microbiota changes under *C. butyricum* supplementation.

## 5. Conclusions

This study demonstrates that administering *C. butyricum* at 5 g/d significantly increased average daily gain (ADG) and reduced diarrhea incidence in preweaning calves, particularly during the first 42 days. Calves in the 5 g/d group exhibited elevated plasma IgM concentrations and T-AOC, concomitant with reduced MDA and IL-1β levels, suggesting enhanced immunocompetence and attenuated oxidative stress. The beneficial effects of *C. butyricum* were attributable to its modulation of the gut microbiota and enhancement of SCFA production. Supplementation with *C. butyricum* at 5 g/d significantly increased the abundance of putative beneficial genera (*Bacteroides*, *Bifidobacterium*, and *Faecalibacterium*) and elevated fecal propionic and butyric acid concentrations at Day 42. On the basis of these findings, we recommend supplementing preweaning calf diets with 5 g/d *C. butyricum* to optimize health and productivity during this critical developmental phase.

## Figures and Tables

**Table 1:** Composition and nutritional level of starter and alfalfa hay (%).

Items	Starter	Alfalfa
Ingredients, %		
Corn	55.10	-
Soybean meal	18.60	-
Corn gluten meal	10.00	-
DGGS	13.00	-
Limestone	1.70	-
NaCl	0.60	-
Premix (1)	1.00	-
Nutrient composition, %		
Dry matter	87.33	91.33
Crude protein	19.72	16.72
Ether extract	4.64	1.56
Ash	5.38	8.17
Neutral detergent fiber	16.53	49.44
Acid detergent fiber	6.02	33.93
Ca	1.15	1.33
P	0.58	0.25

Note: (1) The premix provides the following per kg of the starter diet: VA, 15,000 IU; VD 5000, IU; VE, 50 mg; Fe, 90 mg; Cu, 12.5 mg; Mn, 30 mg; Zn, 90 mg; Se, 0.3 mg; 11.0, mg; Co, 0.5 mg.

**Table 2:** The growth performance and incidence of diarrhea in control calves and calves fed with *C. butyricum*.

Items	Treatment				SEM	*p*-Value
	CON	1 g	3 g	5 g		
Initial BW, kg	40.67	40.44	40.71	40.56	2.654	0.273
Final BW, kg	82.77	85.01	85.51	88.84	2.812	0.161
ADG, g/d	701.67 ^b^	742.83 ^ab^	746.67 ^ab^	804.67 ^a^	28.44	0.040
Starter intake, g of DM/d	23.10	28.47	27.98	30.41	5.016	0.523
Total feed intake, g of DM/d	1231.16	1281.04	1288.77	1302.60	8.121	0.523
Feed efficiency, kg of DMI/kg of gain	1.75	1.72	1.73	1.62	0.023	0.833
Incidence of diarrhea (Days 1 to 21), %	20.91	17.69	15.48	12.33	0.079	0.075
Incidence of diarrhea (Days 22 to 42), %	5.24 ^a^	3.29 ^a^	3.69 ^a^	1.59 ^b^	0.016	0.039
Incidence of diarrhea (Days 43 to 60), %	1.12	1.13	-	-	0.009	0.084

^a,b^ Means in the same row with different superscripts are significantly different (*p* ≤ 0.05).

**Table 3:** The plasma immunoglobulin, cytokine, and antioxidant index concentrations in control calves and calves fed with *C. butyricum*.

Items	Treatment				SEM	*p*-Value
	Con	1 g	3 g	5 g		
IgA, μg/mL						
21 d	190.556	191.437	197.874	218.361	9.55	0.359
42 d	193.604	203.705	211.753	216.104	11.94	0.483
60 d	195.187	187.155	201.536	213.853	10.2	0.288
IgG, μg/mL						
21 d	1904.875	2108.836	2162.57	2178.854	94.56	0.159
42 d	1959.556	2103.27	2248.133	2275.524	110.32	0.254
60 d	2086.701	2032.398	2182.325	2150.534	72.38	0.383
IgM, μg/mL						
21 d	131.158	136.441	143.427	141.858	5.41	0.598
42 d	137.292 ^b^	145.163 ^ab^	141.593 ^ab^	148.927 ^a^	3.21	0.032
60 d	139.627	134.684	142.073	140.109	6.32	0.149
IL-1β, ng/L						
21 d	49.097	48.928	48.448	47.363	2.57	0.269
42 d	52.534 ^a^	51.676 ^ab^	48.828 ^ab^	46.385 ^b^	3.96	0.036
60 d	52.535	49.076	48.846	48.946	2.15	0.365
IL-2, ng/L						
21 d	387.501	382.243	396.41	403.77	19.45	0.613
42 d	403.425	396.398	409.63	423.397	32.28	0.357
60 d	390.366	371.807	398.174	409.329	21.22	0.288
TNF-α, ng/L						
21 d	300.834	299.167	293.484	298.382	15.05	0.873
42 d	314.88	315.263	303.297	299.74	13.1	0.091
60 d	314.863	298.86	293.086	288.66	19.42	0.343
MDA, nmol/mL						
21 d	3.127 ^a^	2.995 ^a^	2.856 ^ab^	2.62 ^b^	0.25	0.033
42 d	3.176 ^a^	3.122 ^a^	2.992 ^ab^	2.825 ^b^	0.23	0.018
60 d	3.038	3.06	2.822	2.823	0.24	0.133
T-AOC, μmol/mL						
21 d	65.79	64.723	70.461	71.638	3.53	0.318
42 d	59.498 ^b^	65.377 ^ab^	68.988 ^a^	72.290 ^a^	2.94	0.003
60 d	68.711 ^b^	69.477 ^ab^	68.636 ^ab^	75.575 ^a^	2.38	0.033

^a,b^ Means in the same row with different superscripts are significantly different (*p* ≤ 0.05).

**Table 4:** Fecal short-chain fatty acid content in control calves and calves fed with *C. butyricum* for 21 days.

Items	Treatment		SEM	*p*-Value
	CON	5 g		
Acetate, μg/g	3001.94	2658.38	179.703	0.368
Propionic acid, μg/g	1307.89	1513.08	132.627	0.466
Isobutyric acid, μg/g	203.45	314.14	56.076	0.348
Butyrate, μg/g	962.71	1179.29	121.704	0.399
Isovaleric acid, μg/g	186.02	304.58	57.388	0.324
Valeric acid, μg/g	143.10	98.02	43.556	0.628
Caproic acid, μg/g	4.97	1.92	1.277	0.25
Total SCFA, μg/mL	5810.08	5792.21	514.052	0.987

**Table 5:** Fecal short-chain fatty acid content in control calves and calves fed with *C. butyricum* for 42 days.

Items	Treatment		SEM	*p*-Value
	CON	5 g		
Acetate, μg/g	5810.08	5792.21	514.052	0.078
Propionic acid, μg/g	1211.59 ^b^	1732.94 ^a^	136.799	0.046
Isobutyric acid, μg/g	1211.59	1691.47	138.301	0.081
Butyrate, μg/g	782.2 ^b^	1197.94 ^a^	108.358	0.04
Isovaleric acid, μg/g	289.91	448.56	65.551	0.244
Valeric acid, μg/g	273.45	340.30	76.251	0.682
Caproic acid, μg/g	2.70	3.13	0.696	0.775
Total SCFA, μg/mL	5760.36	7885.69	611.363	0.080

^a,b^ Means in the same row with different superscripts are significantly different (*p* ≤ 0.05).

**Table 6:** Fecal short-chain fatty acid content in control calves and calves fed with *C. butyricum* for 60 days.

Items	Treatment		SEM	*p*-Value
	CON	5 g		
Acetate, μg/g	2015.03	2401.64	207.547	0.377
Propionic acid, μg/g	896.76	1023.94	123.294	0.629
Isobutyric acid, μg/g	159.61	161.54	28.744	0.975
Butyrate, μg/g	580.64	585.92	75.729	0.947
Isovaleric acid, μg/g	151.31	132.54	32.030	0.785
Valeric acid, μg/g	135.82	108.03	33.584	0.699
Caproic acid, μg/g	3.97	1.24	1.112	0.239
Total SCFA, μg/mL	3943.15	4377.55	445.772	0.652

## Data Availability

The original data presented in the study are openly available in the NCBI SRA under accession PRJNA1294751.
